# Effects of Polarized Training on Cardiometabolic Risk Factors in Young Overweight and Obese Women: A Randomized-Controlled Trial

**DOI:** 10.3389/fphys.2018.01287

**Published:** 2018-09-18

**Authors:** Rafael Zapata-Lamana, Carlos Henríquez-Olguín, Carlos Burgos, Roberto Meneses-Valdés, Igor Cigarroa, Claudio Soto, Valentín E. Fernández-Elías, Sonia García-Merino, Rodrigo Ramirez-Campillo, Antonio García-Hermoso, Hugo Cerda-Kohler

**Affiliations:** ^1^Escuela de Educación, Universidad de Concepción, Los Ángeles, Chile; ^2^Unidad de Fisiología Integrativa, Laboratorio de Ciencias del Ejercicio, Clínica MEDS, Santiago, Chile; ^3^Escuela de Kinesiología, Facultad de Salud, Universidad Santo Tomás, Los Ángeles, Chile; ^4^Department of Sport Science, European University of Madrid, Madrid, Spain; ^5^Laboratorio de Medición y Evaluación Deportiva, Núcleo de Investigación en Salud, Actividad Física y Deporte, Departamento de Ciencias de la Actividad Física, Universidad de Los Lagos, Osorno, Chile; ^6^Laboratorio de Ciencias de la Actividad Física, el Deporte y la Salud, Facultad de Ciencias Médicas, Universidad de Santiago de Chile, Santiago, Chile

**Keywords:** exercise, training modalities, obesity, cardiorespiratory fitness, metabolism

## Abstract

**Introduction:** Volume and intensity are major variables governing exercise training-mediated beneficial effects in both athletes and patients. Although polarized endurance training optimizes and maximizes physiological gains in highly trained individuals, its cardiometabolic protective-effects have not been established. The purpose of the present single site, randomized-controlled trial was to compare the effects of 12-weeks of high-intensity interval training (HIIT), moderate-intensity continuous training (MICT), and polarized volume training (POL) programs on cardiometabolic risk factors in young overweight and obese women.

**Materials and Methods:** A total of 64 overweight/obese young women (age 23.3 ± 3.8 years, body mass index 33.8 ± 3.8 kg/m^2^) were randomly assigned to four groups: control group (CTRL), polarized volume training group, moderate-intensity endurance training group, and HIIT group. The cardiorespiratory capacity, glycemic and lipid profiles, whole-body substrate utilization, and body composition were assessed before and after the intervention.

**Results:** After the intervention, VO_2peak_ and power output at VO_2peak_ increased in all exercised-groups (time effect: *p* < 0.0001). Power output at VT1 was increased only in the POL group compared to the CTRL group (*p* = 0.019). Relative fold changes in fasting plasma glucose concentrations decreased only in POL group (*p* = 0.002). Training induced a significant increase in relative fat oxidation in all the groups (time effect: *p* < 0.001). Relative fat oxidation increased only in the POL group compared to the CTRL group (training effect: *p* = 0.032).

**Conclusion:** Twelve-weeks of polarized volume training showed overall superior effects on cardiorespiratory fitness, basal glycemic control, and substrate oxidation in comparison to MICT and HIIT training modalities. These data suggest that polarized volume training is an effective non-pharmacological treatment strategy for reducing cardiovascular disease risk factors in young overweight and obese women. The trial is registered at ISRCTN, number ISRCTN34421723.

## Introduction

Overweight and low cardiorespiratory fitness are independent risk factors associated with the development of chronic diseases and increased all-cause mortality (Wei et al., 2000; Myers et al., 2015; Ross et al., 2016). In developing countries, over-nutrition and physical inactivity are generally more prevalent among girls and women than their male counterparts (Case and Menendez, 2009; Kanter and Caballero, 2012). In woman, a high cardiorespiratory fitness ameliorates obesity-related comorbidities such as insulin resistance, dyslipidemia, and hypertension (Stevens et al., 2002; Evenson et al., 2004; Hu et al., 2004). Clinical training prescription is one of the most important non-pharmacological interventions to reverse poor cardiorespiratory fitness and improve metabolic health in women with obesity (Baillot et al., 2014; Pedersen and Saltin, 2015).

Training-stimulated physiological benefits are primarily dependent on the volume and intensity of the training stimulus (Dudley et al., 1982; Gormley et al., 2008). A substantial number of research studies have compared the effects of short bouts of high-intensity interval training (HIIT) and long bouts of moderate-intensity continuous endurance training (MICT) in athletes (Laursen, 2010; Ní Chéilleachair et al., 2017), healthy and clinical populations (Kessler et al., 2012; Mora-Rodriguez et al., 2014, 2016). Although the great debate comparing HIIT vs. MICT in training-induced health benefits, the evidence from athletes suggest that both high- and moderate-intensity training are required to maximize the physiological effect of training (Tønnessen et al., 2014; Stöggl and Sperlich, 2015).

The optimal training distribution reported in athletes implies training in accordance with the ∼80–20 rule, i.e., ∼70–80% of training volume near the lactate/ventilatory threshold intensity (i.e., MICT) and ∼20–30% of training volume near the VO_2 max_ (i.e., HIIT) (Tønnessen et al., 2014). In fact, compared to HIIT or MICT modalities alone, polarized training (POL) induces greater improvements in major endurance performance variables (e.g., VO_2 max_, lactate threshold) in well-trained athletes (Stöggl and Sperlich, 2014).

Given the current evidence is still in debate about the superior effects of HIIT or MICT in metabolic benefits of training in obese women (Keating et al., 2014, 2017; Wewege et al., 2017), POL training emerges as an alternative to improve the training-mediated outcomes in clinical populations. However, no studies have addressed the effect of a POL intervention in physical and metabolic health in non-athlete population.

The purpose of the present study was to compare the effects of 12-weeks of HIIT, MICT, or POL training programs on cardiorespiratory fitness, glucose, and lipids homeostasis, whole-body energy substrate oxidation, and body composition in young overweight and obese women. We hypothesized that, similar to athletes (Stöggl and Sperlich, 2014), greater training-induced adaptive responses would be observed after POL compared to either MICT or HIIT protocols.

## Materials and Methods

### Participants Characteristics

The study took place at the facilities from Universidad de Concepción, Concepción, Chile, from May 2015 to December 2015 and the trial was retrospectively registered with http://www.isrctn.com in June 2016. Eighty women responded to the study invitation. Background information, medical history, family history of disease, and current health status were collected via self-reported survey questionnaires (Shephard, 2015).

The following criteria for inclusion were applied: age between 20 and 40 years, body mass index (BMI) between 25 and 40 kg/m^2^, untrained (i.e., <2 h of physical activity/week), and being non-diabetic. Exclusion criteria were family history of stroke, hypertension, cardiovascular or respiratory disease, acute or chronic inflammatory diseases, digestive system surgery, use of thyroid hormone replacement, antidepressant consumption, pregnancy, and recent participation (i.e., less than 2 months) in training or diet interventions. After signing a written informed consent, 64 eligible young women were enrolled in the study. The Ethical Committee from Universidad de Concepción approved the study protocol (Number 2016-PI215136015-1.0IN), and all procedures were performed in compliance with the Declaration of Helsinki for human experiments. The authors confirm that all ongoing and related trials for this intervention are registered.

### Study Design

**FIGURE 1:**
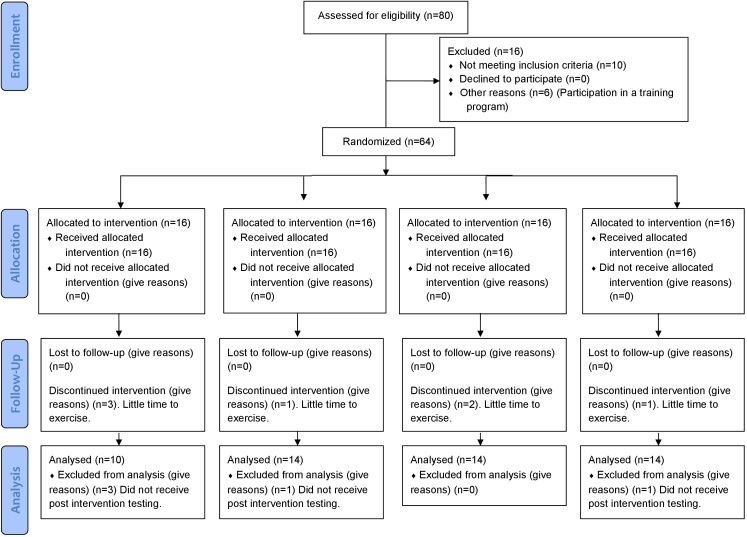
CONSORT flowchart of the study.

The study was a 12-week, single site, parallel-group, randomized-controlled trial, designed to compare the effects of three training protocols on cardiorespiratory and cardiometabolic risk factors in young overweight and obese women. Participants were randomly assigned to four groups: control (CTRL, *n* = 16), polarized-based volume training (POL, *n* = 16), moderate-intensity endurance training (MICT, *n* = 16), and HIIT (*n* = 16) (**Figure 1**). The randomization process was performed using STATA 13.0 (StataCorp, College Station, TX, United States).

### Pre–Post Testing

Participants (**Table 1**) were asked to arrive at the facilities between 07:00 and 10:00 in four different occasions before and after the intervention, with a minimum of 2 days between each visit. Participants were instructed to arrive the laboratory by minimal physical activity, well hydrated, and avoid any moderate-to-vigorous physical exercise for at least 48 h before testing. The sequence of the measurements was: (i) glycemic and lipid homeostasis, (ii) body composition, (iii) cardiorespiratory fitness assessment, and (iv) cycling trial at 60% of pVO_2peak_. A standardized diet (60% carbohydrate, 25% fat, and 15% protein) was prescribed by a dietitian for 1 week before baseline and post-training measurements.

**Table 1:** Basal characteristics before 12-weeks of intervention.

	CTRL	POL	MICT	HIIT
*Age (years)*	22.7 ± 3.2	21.8 ± 1.9	21.3 ± 1.4	21.2 ± 1.4
*BMI (kg.m^-2^)*	33.2 ± 4.2	31.9 ± 2.0	32.2 ± 4.1	31.9 ± 3.0
*HOMA-IR (units)*	4.5 ± 1.9	4.3 ± 1.1	4.5 ± 1.4	4.4 ± 1.9
*VO_2peak_ (mL.kg^-1^.min^-1^)*	25.0 ± 4.0	24.5 ± 2.5	22.7 ± 3.1	25.3 ± 2.6

*Data are shown as mean ± SD*.

### Cardiorespiratory Fitness Assessment

After a 5-min warm-up at 50 watts, the participants performed a maximal incremental test on a cycle-ergometer (Monark, Stockholm, Sweden) according to criteria previously described (Bentley et al., 2007). An initial workload of 55 watts (W) was used, with increments of 15 W every 2 min until exhaustion. Gas exchange was recorded continuously with a portable breath-to-breath gas analyzer (Cortex Metalyzer 3B, Leipzig, Germany). The analyzer was calibrated according to the manufacturer’s instructions before each trial. Pulmonary ventilation (VE), oxygen uptake (VO_2_), expired carbon dioxide (VCO_2_), and respiratory exchange ratio (RER) were averaged over 10 s in the mixing chamber mode, with the highest 30 s value (i.e., three consecutive 10 s averages) used in the analysis. VO_2peak_ was determined according to previously established criteria (Howley et al., 1995): (i) plateau in VO_2_ (i.e., increase <150 ml min^-1^), (ii) RER > 1.1, and (iii) ≥90% of theoretical maximal heart rate. The VO_2 max_ was expressed both as absolute values (L.min^-1^) and relative to body mass (ml.kg^-1^.min^-1^). The power output at VO_2peak_ (pVO_2peak_) was determined as the minimum workload at which VO_2peak_ was reached. The ventilatory threshold 1 (VT1) was determined by the V-slope method (Beaver et al., 1986) and expressed in terms of power output at VT1 (pVT1).

### Glycemic and Lipid Homeostasis

Participants were asked to arrive at the laboratory after an overnight fast and to abstain from moderate-to-vigorous physical exercise for at least 48 h before testing. Blood samples were drawn from an antecubital vein in resting subject for measurements in serum and plasma. Whole-body insulin sensitivity was estimated by an oral glucose tolerance test. For this test, subjects consumed a 75-g oral glucose load within 5 min. After that, blood samples were collected at 30 and 120 min for measurements of plasma glucose and insulin. Blood was immediately centrifuged to isolate and collect serum, and frozen at -80°C until analysis. Serum glucose (mg.dL^-1^) assays were performed on an automated glucose analyzer (Sirrus analyzer; Stanbio Laboratory, Boerne, TX, United States), and serum insulin (μU.ml^-1^) was measured using an immunofluorescent method with an AIA-600 II analyzer (Tosoh Bioscience, South San Francisco, CA, United States) according to the manufacturer’s instructions. Whole-body insulin sensitivity was estimated from the oral glucose tolerance test data using the modified insulin sensitivity index (ISI composite) (DeFronzo and Matsuda, 2010), calculated as *k*/sqrt (G_0_ × I_0_ × G_120_ × I_120_), where *k* (=10,000) is a constant, G_0_ and G_120_ represent the plasma glucose concentrations at times 0 and 120 min, I_0_ and I_120_ represent the plasma insulin levels at times 0 and 120 min, and sqrt is the mathematical function to calculate the square root. Insulin resistance was estimated using the Homeostasis Model for Assessment of Insulin Resistance (HOMA-IR), calculated as [fasting insulin (μU.ml^-1^) × fasting glucose (mmol.L^-1^)]/22.5 (Matthews et al., 1985). Total cholesterol, lipoprotein cholesterol [low-density lipoprotein (LDL), high-density lipoprotein (HDL), very-low-density lipoprotein (vLDL)] and triglycerides were determined via standard enzymatic-colorimetric assays run on a Cobas^®^ 8000 modular analyzer series (Roche Diagnostic Corporation, Indianapolis, IN, United States). LDL and vLDL cholesterol were calculated from total cholesterol, HDL and triglyceride concentrations by applying the Friedewald’s equation.

### Whole-Body Substrate Utilization, Blood Lactate, Heart Rate, Pulmonary Ventilation, and the Rating of Perceived Exertion During Cycling Trial at 60% of pVO_2peak_

The submaximal cycle testing was performed ≥2 days after the maximal incremental test. All subjects arrived for testing between 7:00 and 10:00 am after a 12-h overnight fast. Participants cycled at a constant cadence (i.e., 70–80 rpm) on a bicycle-ergometer (Monark, Stockholm, Sweden) for 30 min at 60% of pVO_2peak_ (according to pre-training or post-training values). Gas exchange measurements were used to calculate whole-body lipid and carbohydrate oxidation according to the following equations (Jeukendrup and Wallis, 2005):

$$\text{Carbohydrate oxidation (g}{\text{.min}}^{-1}\text{): 4}{\text{.585(VCO}}_{2}\text{(L/ min))}-3.226({\text{VO}}_{2}(\text{L}/\;\min))$$

$$\text{Lipid oxidation (g}{\text{.min}}^{-1}\text{): 1}{\text{.695(VO}}_{2}\text{(L/ min))}-1.701({\text{VCO}}_{2}(\text{L}/\;\min)$$

The comparison of substrate oxidation in the 30-min cycling trial was measured at 60% of pVO_2peak_ of pre-training values and post-training values (Post-1) as previously described (Talanian et al., 2007). An additional trial was performed, adjusting the 60% of pVO_2peak_ according to the maximal cardiorespiratory fitness after the training period (Post-2). Blood lactate values were obtained via a finger prick capillary blood sample immediately after the exercise trial. Samples were analyzed immediately for whole blood lactate concentration (mmol/l) using a standard enzymatic lactate analyzer (Venus, HP-Cosmos, Germany). For heart rate (bpm) measurements, a cardiac monitor (RS200; Polar, Kempele, Finland) was used, and the rating of perceived exertion (RPE) was evaluated and recorded immediately after exercise trial using the 10-point Borg scale (Borg, 1982). The participants were instructed on how to use the 10-point Borg scale and accustomed to the scale in the third visit (see section “Pre–Post Testing” for details). Determination of the RPE was assessed according to the AHA Scientific Statement (Myers et al., 2009). To evaluate and record the RPE, the participants were shown the scale and were asked “How would you rate your effort?”

### Body Composition

Total body mass and regional estimates of bone mass, bone mineral density, fat mass, lean mass, and body-fat % were determined by dual X-ray absorptiometry (DXA) using manufacturer-supplied algorithms (Total Body Analysis, version 3.6; Lunar, Madison, WI, United States) as previously described (Ramírez-Campillo et al., 2013). Subjects dressed in underwear and laid face-up on a DXA scanner table. Scanning was performed in 1-cm slices from head to toe using a 20-min scanning speed. The boundary between the arms and trunk was vertical (at shoulder level), whereas the boundary between the legs and trunk was angled. The precision of the measurement reported in these three regions is 1.5, 0.8, and 1.1% for the arms, legs, and trunk, respectively (Nindl et al., 2000).

### Exercise Training Protocols

**FIGURE 2:**
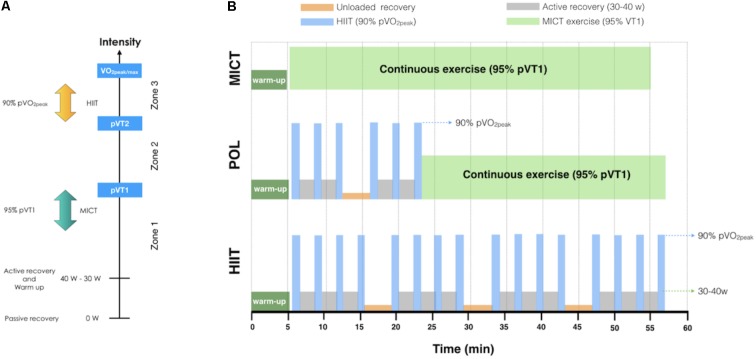
Exercise intensity and training protocols. **(A)** Training zones and intensities used in the exercise training protocols, **(B)** Schematic representation of training protocols. VO_2peak/max_: Peak or maximal oxygen uptake; pVT2: power at ventilatory threshold 2; pVT1: power at ventilatory threshold 1; POL: polarized volume training; MICT: moderate-intensity continuous training; HIIT: high-intensity interval training; W: watts.

The training intervention consisted of 36 cycling sessions spread over 12 weeks (3 sessions/week) supervised by a professional trainer (**Supplementary Table S1**). All training sessions were carried out in the University facilities under conditions of standard temperature (21 ± 1°C) and humidity (50 ± 10%). After a 5-min warm up, the experimental groups performed the following training protocols (**Figure 2**).

#### CTRL Group

No training intervention was performed; the participants were instructed not to partake in any formal physical activity and diet modification.

#### POL Group

We adapted the previously described protocols for a clinical setting (Stöggl and Sperlich, 2015). In order to maintain the polarized pattern, the training protocol for the POL group aimed to conduct 70–80% of the total training volume at low-moderate intensity and 20–30% at high-intensity (Stöggl and Sperlich, 2014). For practical reasons, the moderate and high-intensity protocols were performed in every session, and not in a block periodization pattern as in athlete population (Stöggl and Sperlich, 2014). Each session consisted of 30 min of cycling exercise at a constant cadence (70–80 rpm) at 95% of pVT1; plus two sets of three bouts of 60 s cycling exercise at 90% pVO_2peak_, with 2 min of active recovery between bouts (∼30–40 W) and 4 min of unloaded recovery (backward pedaling on the cycle-ergometer) between sets.

#### MICT Group

The continuous training was performed according to a previously reported threshold model (Stöggl and Sperlich, 2014). Each training session consisted of 45–50 min of cycling exercise at a constant cadence (70–80 rpm) at 95% of pVT1.

#### HIIT Group

Four sets of four bouts of 60 s cycling exercise at 90% pVO_2peak_, with 2 min of active recovery between bouts (∼30–40 W) and 4 min of unloaded recovery (backward pedaling on the cycle-ergometer) between sets.

These protocols remained constant for every training session. The RPE was evaluated at the end of each training session. If the RPE (1 point) and mean heart rate (5–10 bpm) were decreased for two consecutive training sessions, then the power output for the intervals was increased by 5–10 W to maintain training overload. For heart rate and RPE assessment we used the same methods as described above. Caloric intake was not standardized or controlled during the training intervention, but all the participants were instructed to maintain their regular dietary habits throughout the study.

### Statistical Analysis

All data were expressed as mean ± standard deviation (SD) or 95% confidence interval (CI), unless otherwise stated. Normality and sphericity assumptions were checked with the Shapiro–Wilk test and epsilon coefficient, respectively. Sphericity was corrected with the Greenhouse-Geisser correction method. If the normality assumption was violated, data were log-transformed to reduce bias arising from non-uniformity error (Hopkins et al., 2009). This was the case for relative carbohydrate oxidation, total carbohydrate oxidation, and total fat oxidation. The data were analyzed using mixed factorial ANOVAs, which included the between-subjects factor of training model (CTRL, POL, MICT, and HIIT groups) and the within-subjects factor of time (pre-training versus post-training, i.e., repeated measured analysis) to identify significant main and interaction effects. A Bonferroni’s *post hoc* test for multiple comparisons was performed if a significant main effect was observed. Statistical significance was set at *p* < 0.05. Sample size (*n* = 14 per group) allows detection of a 37 watts change in pVO_2peak_, which is in agreement with the study of Keating et al. (2014) with an alpha error of 0.05 and a power of 80%. As a complementary analysis, we assessed the magnitude of the effect of the intervention (within-subjects factor) via estimation of the size of an effect (ES) (Ialongo, 2016). Because the formula for Cohen’s *d* gives a biased estimate of the ES, especially for small samples (*n* < 20), the magnitudes of change after training were calculated through the *corrected effect size* (Hedges’s *g*, with 95% CI) (Lakens, 2013). The following threshold values for ES were employed: <0.2 as trivial, ≥0.2 as small, ≥0.6 as moderate, ≥1.2 as large, ≥2.0 as very large, and ≥4.0 as extremely large (Stanley et al., 2015). If the confidence interval overlapped thresholds for substantial positive and negative values, the effect was deemed unclear (i.e., trivial). The correlation between variables was analyzed by Pearson’s correlation coefficient (r). To construct confidence intervals for ES and r that does not depend on normality assumption, confidence intervals were calculated with non-parametric bootstrap according to Carpenter and Bithell (2000). A minimum training compliance of 80% was required to be included in the final statistical analysis. Statistical analysis was performed using STATA 13.0 (StataCorp, College Station, TX, United States).

## Results

We recruited 80 participants, of which 28 were excluded for various reasons (see CONSORT flowchart in **Figure 1**). The final analysis, therefore, included 52 patients, 10 in the CTRL group, 14 in the POL group, 14 in the MICT group, and 14 in the HIIT group.

### Cardiorespiratory Fitness

**FIGURE 3:**
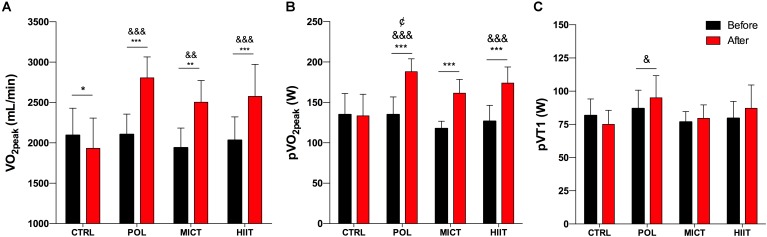
Effects of training intervention on cardiorespiratory capacity. **(A)** Peak oxygen uptake (VO_2peak_), **(B)** power at VO_2peak_ (pVO_2peak_), and **(C)** power at VT1 (pVT1) before and after 12-weeks of control (CTRL), polarized volume training (POL), moderate-intensity endurance training (MICT), and high-intensity interval training (HIIT). Data are shown as mean ± SD. ^∗^, ^∗∗^, and ^∗∗∗^ denote differences between pre and post-training values (*p* < 0.05, *p* < 0.01, and *p* < 0.001, respectively). ^&^, ^&&^, and ^&&&^ denote differences vs. CRTL group after intervention (*p* < 0.05, *p* < 0.01, and p < 0.001, respectively). cDenote differences between POL and MICT groups after intervention (*p* < 0.05). The comparison was performed with Mixed Factorial ANOVAs with Bonferroni’s *post hoc* test for multiple comparisons.

Before exercise intervention, no differences were observed between groups for VO_2peak_, pVO_2peak_, or pVT1 (**Figures 3A–C**, respectively). After the intervention, VO_2peak_ increased significantly in all exercised-groups (time effect: *p* < 0.0001) with a very large ES for POL (g = 2.6 [1.5/3.8, 95% CI]), and for MICT (g = 2.1 [0.9/3.3, 95% CI]), and a large ES for the HIIT group (g = 1.5 [0.6/2.3, 95% CI]). As shown in **Figure 3B**, a significant increase in pVO_2peak_ was found for all exercised-groups (time effect: *p* < 0.0001) with a very large ES for POL (g = 2.7 [1.5/3.9, 95% CI]), for MICT (g = 3.0 [1.4/4.7, 95% CI]), and for the HIIT group (g = 2.3 [1.2/3.3, 95% CI]). When comparing exercise-groups, POL was significantly superior vs. MICT group at increasing pVO_2peak_ (training effect: *p* = 0.029). Regarding pVT1 (**Figure 3C**), the values were significantly increased only in the POL group compared to the CTRL group (*p* = 0.019).

### Blood Glucose, Insulin, and Lipid Profile

**FIGURE 4:**
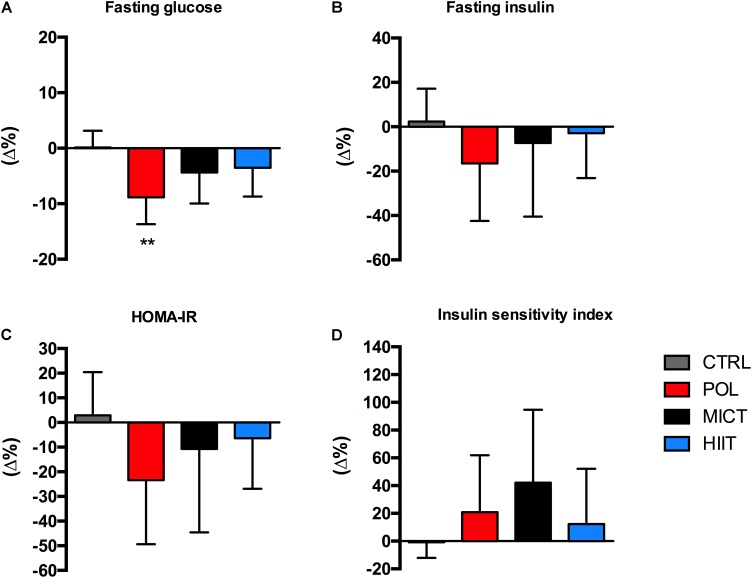
Effects of training intervention on glycemic control. **(A)** Fasting plasma glucose, **(B)** fasting plasma insulin, **(C)** HOMA-IR, and **(D)** insulin sensitivity index before and after 12-weeks of control (CTRL), polarized volume training (POL), moderate-intensity endurance training (MICT), and high-intensity interval training (HIIT). Data are shown as mean ± SD of relative fold changes (Δ%). ^∗∗^Denote differences between pre and post-training values (*p* < 0.01). The comparison was performed with Mixed Factorial ANOVAs with Bonferroni’s *post hoc* test for multiple comparisons. HOMA-IR, homeostasis model for assessment of insulin resistance.

To study training-induced changes in glucose homeostasis, we measured fasting glucose and insulin before and after the intervention (**Supplementary Table S2**). As shown in **Figure 4A**, the relative change in fasting plasma glucose concentration decreased only significantly in the POL group (*p* = 0.002) with a large ES for POL (g = -1.37 [-2.2/-0.4, 95% CI]) and an unclear ES for MICT, HIIT, and CTRL group. Fasting plasma insulin (**Figure 4B**) showed a moderate ES for POL (g = -1.10 [-2.0/-0.1, 95% CI] whereas no changes were found for HOMA-IR (**Figure 4C**) or insulin sensitivity index (**Figure 4D**).

No differences were observed between the groups at baseline in any plasma lipid parameter (**Table 2**). After training, total cholesterol was not modified by any training modality. HDL decreased significantly in the HIIT group (time effect: *p* = 0.013). Interestingly, LDL and plasma triglycerides decreased significantly in the POL group but not in the MICT or HIIT groups (time effect: *p* = 0.007 and *p* = 0.017, respectively).

**Table 2:** Changes in plasma lipids after 12-weeks of intervention.

	Pre	Post (Δ vs. Pre)	Normal-based ES (95% CI)
**Total cholesterol (mg.dL^-1^)**			
CTRL	178.5 ± 26.5	0.1 ± 2.9	0.01 (-1.0/1.0)
POL	177.9 ± 32.0	-9.7 ± 23.8	-0.26 (-1.2/0.7)
MICT	172.5 ± 36.1	-7.9 ± 19.2	-0.19 (-1.3/0.9)
HIIT	165.4 ± 29.5	-7.6 ± 19.2	-0.26 (-1.1/0.6)
**HDL-cholesterol (mg.dL^-1^)**			
CTRL	54.8 ± 10.6	0.6 ± 2.5	0.05 (-0.9/1.1)
POL	50.9 ± 13.9	0.9 ± 6.5	0.05 (-0.8/0.9)
MICT	52.2 ± 11.5	-1. ± 6.0	-0.07 (-1.2/1.1)
HIIT	56.9 ± 12.7	-3.9 ± 3.6*	-0.28 (-1.1/0.6)
**LDL-cholesterol (mg.dL^-1^)**			
CTRL	100.3 ± 20.8	0.1 ± 2.9	0.01 (-1.0/1.0)
POL	104.3 ± 27.3	-14.2 ± 16.0**	-0.58 (-1.5/0.3)
MICT	98.5 ± 26.2	-9.4 ± 23.7	-0.35 (-1.5/0.8)
HIIT	85.0 ± 26.7	-7.2 ± 14.4	-0.30 (-1.1/0.5)
**Cholesterol ratio (Total/HDL)**			
CTRL	3.3 ± 0.8	0.0 ± 0.1	0.03 (-1.0/1.0)
POL	3.6 ± 1.0	-0.2 ± 0.5	-0.20 (-0.7/1.1)
MICT	3.3 ± 0.4	-0.1 ± 0.3	-0.16 (-1.0/1.3)
HIIT	3.0 ± 0.7	0.1 ± 0.3	0.14 (-1.0/0.7)
**Triglycerides (mg.dL^-1^)**			
CTRL	101.2 ± 23.2	-0.2 ± 2.7	-0.01 (-0.9/0.9)
POL	114.8 ± 33.7	-15.3 ± 21.1*	-0.42 (-1.5/0.6)
MICT	99.4 ± 32.7	-5.6 ± 9.9	-0.14 (-1.2/1.0)
HIIT	115.2 ± 58.9	4.0 ± 26.6	0.05 (-0.7/0.9)

*Data are shown as mean ± SD. ES, effect size; CI, confidence interval; HDL, high-density lipoproteins; LDL, low-density lipoproteins. ^∗^ and ^∗∗^ denote differences between pre and post-training values (p < 0.05 and p < 0.01, respectively). The comparison was performed with Mixed Factorial ANOVAs with Bonferroni’s post hoc test for multiple comparisons*.

### Whole-Body Substrate Utilization During Cycling at 60% of pVO_2peak_

**FIGURE 5:**
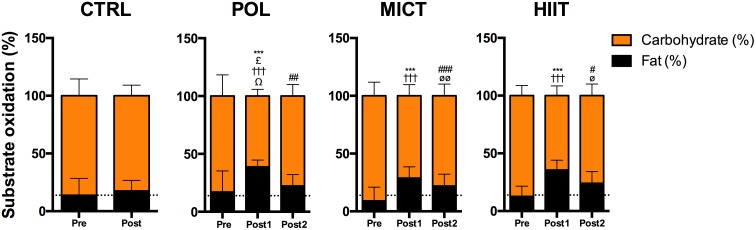
Effects of training intervention on whole-body substrate utilization during a cycling trial at 60% of pVO_2peak_. Relative energy expenditure (%) from fat and carbohydrate oxidation before and after 12-weeks of control (CTRL), polarized volume training (POL), moderate-intensity endurance training (MICT), and high-intensity interval training (HIIT). Data are shown as mean ± SD. ^∗∗∗^Denote differences between Pre and Post-1 values for fat oxidation (time effect: *p* < 0.001). ^£^Denote differences in fat oxidation for Post-1 values between POL vs. CTRL group after intervention (training effect: *p* < 0.05). ^#^, ^##^ and ^###^Denote differences between Pre and Post-2 values for fat oxidation (time effect: *p* < 0.05, *p* < 0.01, and *p* < 0.001, respectively). ^†††^Denote differences between Pre and Post-1 values for carbohydrate oxidation (time effect: *p* < 0.001). ^Ω^Denote differences in carbohydrate oxidation for Post-1 values between POL vs. CTRL group after intervention (training effect: *p* < 0.05). ^øø^ and ^ø^ denote differences between Pre and Post-2 values for carbohydrate oxidation (time effect: *p* < 0.001). The comparison was performed with Mixed Factorial ANOVAs with Bonferroni’s *post hoc* test for multiple comparisons. Post-1: 60% of pVO_2peak_ of pre-training values. Post-2: 60% of pVO_2peak_ according to the cardiorespiratory capacity after training period.

To study the changes in energy substrate utilization during exercise, participants performed 30 min of steady-state exercise at 60% of pVO_2peak_ before and after the intervention (**Figure 5** and **Supplementary Table S2**).

Training induced a significant increase in relative fat oxidation in all groups in Post-1 values (time effect: *p* < 0.001) with a large ES for POL (g = 1.4 [0.5/2.3, 95% CI]), for MICT (g = 1.6 [0.2/3.1, 95% CI]), and for the HIIT group (g = 1.7 [0.8/2.6, 95% CI]). Interestingly, relative fat oxidation increased significantly only in the POL group compared to the CTRL group (training effect: *p* = 0.032). After adjusting the load to the new pVO_2peak_ measured after the intervention (Post-2), the relative fat oxidation increased significantly (time effect: *p* = 0.004, *p* < 0.001, *p* = 0.016; POL, MICT, and HIIT group, respectively) with a moderate ES for the HIIT group (g = 1.0 [0.2/1.9, 95% CI]) and an unclear ES for the POL, MICT, and CTRL groups. Moreover, a significant decrease was found in all exercised-groups in relative carbohydrate oxidation in Post-1 values (time effect: *p* < 0.001) with a large ES for POL (g = -1.4 [-2.6/-0.2, 95% CI]), and for MICT (g = -1.6 [-3.1/-0.2, 95% CI]), and a very large ES for the HIIT group (g = -2.4 [-3.6/-1.3, 95% CI]). Interestingly, only the POL group showed significant differences compared to the CTRL group after intervention (training effect: *p* = 0.017). Relative carbohydrate oxidation decreased significantly for Post-2 values in MICT and HIIT groups (time effect: *p* = 0.008 and *p* = 0.016, respectively) with a moderate ES for HIIT group (g = -1.1 [-2.0/-0.2, 95% CI]).

### Cardiorespiratory Response, Blood Lactate, and RPE During Cycling at 60% of pVO_2peak_

**FIGURE 6:**
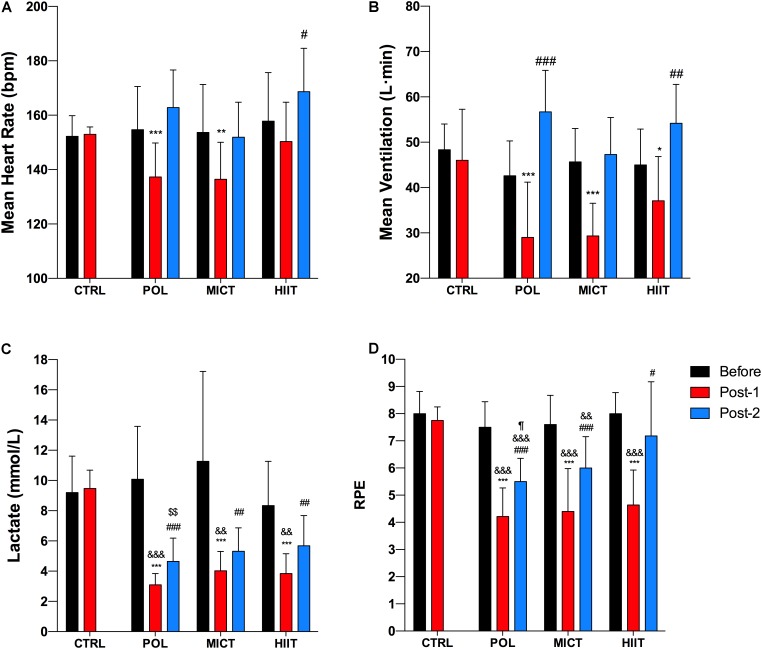
Effects of training intervention on cardiorespiratory response, blood lactate, and rating of perceived exertion during a cycling trial at 60% of pVO_2peak_. **(A)** Mean heart rate, **(B)** mean ventilation, **(C)** blood lactate, and **(D)** RPE after 12-weeks of control (CTRL), polarized volume training (POL), moderate-intensity endurance training (MICT), and high-intensity interval training (HIIT). Data are shown as mean ± SD. ^∗^, ^∗∗^, and ^∗∗∗^ denote differences between Pre and Post-1 values (time effect: *p* < 0.05, *p* < 0.01, and *p* < 0.001, respectively). ^#^, ^##^, and ^###^ denote differences between Pre and Post-2 values (time effect: *p* < 0.05, *p* < 0.01, and *p* < 0.001, respectively). ^&&^ and ^&&&^ denote differences in Post-1 values vs. CTRL group after intervention (*p* < 0.01 and *p* < 0.001, respectively). ^$$^Denote differences in Post-2 values vs. CTRL group after intervention (*p* < 0.01). ^¶^ Denote differences in Post-2 values between Pol and HIIT group after intervention (*p* < 0.05). The comparison was performed with Mixed Factorial ANOVAs with Bonferroni’s *post hoc* test for multiple comparisons. Post-1 and Post-2: after training at 60% of pre-pVO_2peak_ value and 60% of post-pVO_2peak_ value, respectively. RPE, rating of perceived exertion.

Before the intervention, no differences were observed between groups for mean VE, mean HR, blood lactate, and RPE (**Figures 6A–D**, respectively).

After the training period, the mean HR values in Post-1 (**Figure 6A**) showed a significant decrease in the POL and MICT groups (time effect: *p* < 0.001; *p* = 0.001, respectively) with a moderate ES for POL (g = -1.1 [-2.1/-0.1, 95% CI]), and an unclear ES for MICT, HIIT, and CTRL groups. For the Post-2 values, mean HR increased significantly in the HIIT group (time effect: *p* = 0.010) with an unclear ES for all groups.

Mean VE in Post-1 values (**Figure 6B**) decreased significantly in all training groups (time effect: *p* < 0.001; *p* < 0.001; *p* = 0.014; POL, MICT, and HIIT group, respectively) with a large ES for MICT (g = -2.1 [-3.4/-0.8, 95% CI]), a moderate ES for the HIIT group (g = -0.8 [-1.6/-0.07, 95% CI]), and an unclear ES for the POL and CTRL groups. POL and MICT groups showed significant differences compared to the CTRL group after the intervention (training effect: *p* = 0.001 and *p* = 0.002, respectively). For the Post-2 values, the mean VE increased significantly in POL (time effect: *p* < 0.001) and HIIT (time effect: *p* = 0.006) with a large ES for POL (g = 1.5 [0.3/2.8, 95% CI]), and an unclear ES for the MICT and HIIT groups.

After the training period, a significant decrease in blood lactate (**Figure 6C**) was found in all exercised groups in Post-1 values (time effect: *p* < 0.001) with a very large ES for POL (g = -2.6 [-5.1/-0.2, 95% CI]), and a large ES for MICT (g = -1.6 [-2.6/-0.5, 95% CI]), and for the HIIT group (g = -1.9 [-3.1/-0.6, 95% CI]). Additionally, the analysis showed significant differences between the POL, MICT, and HIIT group compared to the CTRL group after the intervention (training model effect: *p* < 0.001, *p* = 0.001, and *p* = 0.001, respectively). For the Post-2 values, blood lactate decreased significantly in all exercised groups compared to baseline (time effect: *p* < 0.001 for POL and MICT group, *p* = 0.004 for the HIIT group) with a very large ES for POL (g = -2.0 [-3.3/-0.6, 95% CI]), a large ES for MICT (g = -1.3 [-2.1/-0.4, 95% CI]), and a moderate ES for the HIIT group (g = -1.0 [-1.8/-0.2, 95% CI]). Interestingly, only POL group showed significant differences compared to the CTRL group after the intervention (training model effect: *p* < 0.001).

After the training period, a significant decrease in RPE (**Figure 6D**) was found in all exercised groups in Post-1 values (time effect: *p* < 0.001) with a very large ES for POL (g = -3.1 [-4.3/-1.9, 95% CI]), for MICT (g = -2.2 [-3.8/-0.6, 95% CI]), and for the HIIT group (g = -3.0 [-4.1/-1.9, 95% CI]), and an unclear ES for the CTRL group. Our results showed significant differences between the POL, MICT, and HIIT groups compared to the CTRL group after the intervention (training model effect: *p* < 0.001). For the Post-2 values, RPE decreased significantly in all exercise-groups (time effect: *p* < 0.001 for POL and MICT group, *p* = 0.018 for HIIT group) with a very large ES for POL (g = -2.1 [-3.1/-1.1, 95% CI]), a large ES for MICT (g = -1.3 [-2.3/-0.2, 95% CI]), and an unclear ES for the HIIT group. Also, the results showed significant differences between the POL (training model effect: *p* < 0.001) and MICT groups (training model effect: *p* = 0.009) compared to the CTRL group after the intervention.

### Changes in Body Weight and Body Composition

Before the intervention, no differences were observed between groups in total body mass, fat mass, and fat-free mass (**Table 3**). Total body mass decreased significantly in POL and MICT but not in the HIIT group (time effect: *p* < 0.001 and *p* = 0.033, respectively). Interestingly, fat mass decreased significantly only in the POL group (time effect: *p* < 0.001).

**Table 3:** Changes in body weight and body composition after 12-weeks of intervention.

	Pre	Post (Δ vs. Pre)	Normal-based ES (95% CI)
**Total body mass (kg)**			
CTRL	84.7 ± 12.8	0.3 ± 1.1	0.02 (-0.9/0.9)
POL	86.6 ± 8.0	-3.7 ± 3.4***	-0.45 (-1.3/0.4)
MICT	82.5 ± 11.6	-1.9 ± 2.6*	-0.14 (-1.2/0.9)
HIIT	84.7 ± 11.9	-0.4 ± 1.4	-0.03 (-0.9/0.8)
**Fat mass (kg)**			
CTRL	38.3 ± 7.1	0.2 ± 0.6	0.02 (-0.9/1.0)
POL	38.4 ± 5.3	-3.0 ± 3.7***	-0.59 (-1.4/0.2)
MICT	37.2 ± 6.8	-1.7 ± 2.8	-0.21 (-1.2/0.8)
HIIT	38.5 ± 7.2	-1.2 ± 1.3	-0.15 (-1.0/0.6)
**Fat-free mass (kg)**			
CTRL	43.4 ± 6.3	0.0 ± 0.5	0.00 (-0.9/0.9)
POL	44.6 ± 3.8	0.2 ± 1.0	0.04 (-0.7/0.8)
MICT	41.3 ± 5.6	1.2 ± 3.9	0.20 (-0.8/1.2)
HIIT	42.8 ± 5.0	1.3 ± 1.2	0.23 (-0.6/1.0)

*Data are shown as mean ± SD. ES, effect size; CI, confidence interval. ^∗^ and ^∗∗∗^denote differences between pre and post-training values (p < 0.05 and p < 0.001, respectively). The comparison was performed with Mixed Factorial ANOVAs with Bonferroni’s post hoc test for multiple comparisons.*

## Discussion

The present study showed for the first time that 12 weeks of polarized volume training is an effective exercise intervention to improve cardiorespiratory fitness and cardiometabolic profile in untrained young overweight and obese women, and further, it leads to greater adaptations in some metabolic health-related outcomes compared to pure MICT and HIIT training regimens. Thus, our data suggest that polarized-based volume training may be a more efficient training method than MICT and HIIT for young overweight and obese women concerning reversing and preventing cardiovascular and metabolic health problems.

Elevated cardiorespiratory fitness is a protective factor against obesity-related metabolic dysfunctions (Hamer and O’Donovan, 2010). Similar adaptations in VO_2max/peak_ have been consistently reported after HIIT and MICT programs in obese/overweight patients (Ortega et al., 2015; Mora-Rodriguez et al., 2016; Batacan et al., 2017; Guadalupe-Grau et al., 2018). To our knowledge, we are the first to apply a polarized volume training program to an untrained and overweight population, and to show that POL training-induced superior cardiorespiratory fitness adaptations compared to MICT and HIIT training. Similar results have been reported in highly trained subjects regarding endurance performance after POL compared with high training volume or high intensity (Stöggl and Sperlich, 2014).

The training-induced changes in body composition without changes in energy intake are controversial. Most of the studies comparing HIIT and MICT have shown consistent small changes in body weight, fat mass, or fat-free mass (Martins et al., 2016; Keating et al., 2017; Wewege et al., 2017). Similarly, our results show no differences between HIIT and MICT in body weight, lean or fat mass (**Table 1**). Interestingly, although POL training-induced changes in body composition did not differ from those induced by HIIT and MICT, it was the only type of exercise that significantly decreased fat mass after the exercise intervention. Nevertheless, a limitation of our study was the lack of dietary control. Participants were instructed to maintain their regular nutritional habits, but no record of food consumption was done. Therefore, although participants that performed POL training lost more weight than the HIIT or MICT groups, it cannot be asserted if POL training-induced more marked changes in body composition.

Exercise training is widely recognized as a therapeutic tool to improve glucose and insulin homeostasis in obese and insulin resistant states (Pedersen and Saltin, 2015). However, the optimal exercise-dose to reduce obesity-induced hyperglycemia and hyperinsulinemia have not been fully established. Previous studies have compared the effect of HIIT compared to MICT insulin sensitive surrogates with contradictory results (Mitranun et al., 2014; Fisher et al., 2015; Ortega et al., 2015; Cocks et al., 2016; De Lorenzo et al., 2018). We found that fasting glucose was reduced only in the POL group, without changes in fasting insulin, insulin sensitivity index or HOMA-IR. These results suggest that dietary intervention in combination with exercise is necessary to improve overall glucose and lipid homeostasis outcomes in obese patients (Christiansen et al., 2010).

Metabolic inflexibility is a key dysfunction in obesity-related diseases (Apostolopoulou et al., 2016; Goodpaster and Sparks, 2017), which is characterized an inability to appropriately switch between glucose and lipids as metabolic substrates. It was recently suggested that low fat oxidation and high plasma lactate levels during exercise are associated with metabolic inflexibility (San-Millán and Brooks, 2018). In the present study, all training programs reduced lactate levels during submaximal exercise (**Figure 6C**). However, in the adjusted exercise trial (Post-2) blood lactate was significantly reduced only in the POL training group compared to the control group. Moreover, POL training increased fat oxidation compared to the control group during Post-1, while MICT and HIIT did not. Thus, it seems that POL training is a more efficient exercise strategy to reduce the metabolic inflexibility present in the overweight population. Regarding the RPE values (**Figure 6D**), all training groups improved this variable during Post-1 and Post-2 exercise after the training period. In addition, when comparing the Post-2 results, the POL group decreased their RPE values compared to the HIIT group, suggesting a superior improvement in exercise tolerance after POL training compared to HIIT, despite HIIT training comprising a greater high-intensity exercise volume. These data are in accordance with previous works comparing HIIT against lower intensity exercise modalities (Astorino et al., 2016; Silva et al., 2017), showing that HIIT training does not reduce RPE during a moderate-intensity exercise. Thus, POL training seems to be more efficient at improving exercise tolerance in untrained overweight women.

As previously reported in training studies, none of the training interventions changed total cholesterol levels (Hagan et al., 1986; Ortega et al., 2013; Guadalupe-Grau et al., 2018). Nevertheless, we found a significant decrease in LDL cholesterol and triglycerides levels after POL training, which may suggest a superior effect of combining MICT and HIIT exercise on lipids profile compared to either MICT or HIIT separately. We speculate that combining both exercise modalities lead to stimulation of both peripheral and central adaptations that together could have a greater impact on the blood lipid profile. Unfortunately, since we did not control food ingestion, we cannot discard dietary modifications as the cause of the decrease in LDL and triglycerides levels. Thus, future POL based exercise interventions controlling caloric ingestion are warranted.

Taken together, the results of our study suggest that polarized training is a novel training modality for patients with metabolic complications. Patients may get benefits performing a large volume of low-intensity exercise plus short intervals of high-intensity bouts to improve cardiometabolic health. Clinical exercise prescription could be simplified using training zones (zones 1, 2, and 3) derived from routinary clinical physiological assessments as previously described in athletes (Seiler and Kjerland, 2006; Stöggl and Sperlich, 2014).

### Limitations and Perspectives

The current study aims to investigate the effect of a POL training compared to MICT and HIIT in metabolic adaptations and cardiovascular risk factors in obese women. Despite our results show the benefits of POL training over HIIT and MICT, some limitations need to be acknowledged. The lack of dietary control limits the conclusions about POL training inducing more substantial weight loss that HIIT and MICT. Although the participants were instructed to maintain their habitual energy intake, the changes in body composition cannot be explained only by the intervention.

The effect of the menstrual cycle in responses to exercise in women has remained controversial. Some (Zderic et al., 2001; Nakamura et al., 2010) but not all (Horton et al., 2002; Vaiksaar et al., 2011) the studies have reported changes in substrate utilization according to the menstrual cycle phase. The current study did not address the menstrual cycle of the participants; therefore we are unable to determine whether the adaptations to the different training protocols can be affected by the menstrual cycle.

The combination of exercise modalities could modify the training-induced outcomes (Coffey and Hawley, 2017). The optimal exercise order to maximize training benefits in clinical populations remains to be further investigated. Moreover, the treatment adherence is a key factor determining training outcomes, and future research should determine the affective and enjoyment responses to POL training compared with other modalities.

## Conclusion

The current study demonstrated that POL training elicits superior cardiorespiratory and metabolic adaptations compared to HIIT or MICT alone, since more profound improvements were found in exercise substrate oxidation and energy efficiency, perceived exertion after exercise, fasting glucose-insulin homeostasis, and the plasma lipid profile in young overweight and obese women.

Our data suggest that combination of HIIT and MICT in a polarized volume distribution could be a more effective exercise regimen compared to HIIT or MICT alone with regards to improving cardiorespiratory fitness and counteracting cardiometabolic risk associated with overweight and obesity.

## Author Contributions

RZ-L, CH-O, and HC-K contributed to the design of the study and drafted the manuscript for publication. RZ-L, RM-V, and CS contributed to data acquisition. CH-O and HC-K contributed to analysis and interpretation of the data. All authors contributed to critically reviewing the manuscript for intellectual content and gave final written approval of the manuscript for publication and agreed to be accountable for the accuracy and integrity of the data.

## Conflict of Interest Statement

The authors declare that the research was conducted in the absence of any commercial or financial relationships that could be construed as a potential conflict of interest.
