# Supplementary material for: Effects of Polarized Training on Cardiometabolic Risk Factors in Young Overweight and Obese Women: A Randomized-Controlled Trial
Source: Front Physiol. 2018 Sep 18;9:1287. doi: 10.3389/fphys.2018.01287 (PMC6153353; doi:10.3389/fphys.2018.01287)
Supplement: Supplementary file 1 [file Table_1.DOCX]

**SUPPLEMENTARY INFORMATION**

**Table S1.** Exercise Training Data.

|  | CTRL | POL | MICT | HIIT |
| --- | --- | --- | --- | --- |
| Protocol | - | MICT: 30 min cycling (70-80 rpm).  HIIT: 2 sets x 3 bouts x 60s cycling.  2 min rest between bouts.  4 min rest between sets. | 45-50 min cycling (70-80 rpm) | 4 sets x 4 bouts x 60s cycling.  2 min rest between bouts.  4 min rest between sets. |
| Frequency | - | 3 sessions per week | 3 sessions per week | 3 sessions per week |
| Intensity | - | MICT: 95% of pVT1.  HIIT: 90% pVO_2peak_  Recovery between bouts: ~30-40 W  Recovery between sets: unload | 95% of pVT1. | 90% pVO_2peak._  Recovery between bouts: ~30-40 W  Recovery between sets: unload |
| Weekly training time | - | MICT: 90 min  Interval: 18 min  Recovery: 12 min  Total: 120 min | 135-150 min | Interval: 48 min  Recovery: 108 min  Total: 156 min |

**Table S2. Changes in substrate oxidation and basal glucose homeostasis after 12-weeks of intervention.** Data are shown as mean ± SD.  ** and *** denote differences between Pre and Post-1 values for fat oxidation, and between Pre and Post values for basal glycemia (time effect: p<0.001). ^Ω^ denote differences in fat oxidation for Post-1 values between POL vs. CTRL group after intervention (training effect: p<0.05). ^###^ denote differences between Pre and Post-2 values for fat oxidation (time effect: p<0.001). ^†††^ denote differences between Pre and Post-1 values for carbohydrate oxidation (time effect: p<0.001). ^Ω^ denote differences in carbohydrate oxidation for Post-1 values between POL vs. CTRL group after intervention (training effect: p<0.05). ^øø^ and ^ø^ denote differences between Pre and Post-2 values for carbohydrate oxidation (time effect: p<0.001). The comparison was performed with Mixed Factorial ANOVAs with Bonferroni´s post hoc test for multiple comparisons. Post-1: 60% of pVO_2peak_ of pre-training values. Post-2: 60% of pVO_2peak_ according to the cardiorespiratory capacity after training period. ES: effect size; CI: confidence interval.

|  | **Pre** | **Post-1** | **Post-2** | | **Normal-based**  **ES Pre vs Post-1 (95% CI)** | **Normal-based**  **ES Pre vs Post-2 (95% CI)** |
| --- | --- | --- | --- | --- | --- | --- |
| **Relative fat oxidation**  **(mg.kgFFM^-1^.min^-1^)** | | | |  | | |
| CTRL | 2.14±1.4 | 2.18±0.8 | ----- | | 0.2 (-2.3/2.7) | ---- |
| POL | 2.57±2.7 | 4.30±1.2^***^ | 4.38±2.0^##^ | | 1.0 (0.05/2.0) | 0.8 (-0.1/1.7) |
| MICT | 1.28±1.6 | 3.22±1.4^**^ | 3.61±1.5^##^ | | 1.3 (-0.07/2.7) | 1.5 (-0.08/3.1) |
| HIIT | 2.05±1.4 | 4.99±1.5^***^ | 4.61±1.7^###^ | | 1.8 (0.8/2.8) | 1.5 (0.5/2.6) |
| **Relative carbohydrate**  **oxidation**  **(mg.kgFFM^-1^.min^-1^)** | | | |  | | |
| CTRL | 36.4±14.9 | 31.3±7.3 | ---- | | -0.2 (-2.2/1.8) | ---- |
| POL | 33.5±10.7 | 16.8±6.8^†††, Ω^ | 37.5±9.0 | | -1.8 (-3.1/-0.6) | 0.4 (-0.3/1.2) |
| MICT | 38.1±10.7 | 19.0±5.4^†††^ | 33.3±10.4 | | -2.1 (-3.7/-0.6) | -0.4 (-1.4/0.6) |
| HIIT | 36.6±8.9 | 22.3±7.2^†††^ | 36.2±8.8 | | -1.6 (-2.5/-0.7) | -0.04 (-0.9/0.8) |
| **Basal glycemia (mg.dL^-1^)** | **Pre** | **Post** |  | |  |  |
| CTRL | 94.7±8.5 | 94.8±9.0 | ---- | | 0.01 (-1.0/1.1) | ---- |
| POL | 95.4±5.8 | 86.9±6.0** | ---- | | -1.3 (-2.2/-0.4) | ---- |
| MICT | 90.6±5.9 | 86.5±6.7 | ---- | | -0.6 (-2.0/0.8) | ---- |
| HIIT | 87.6±5.1 | 84.3±4.1 | ---- | | -0.6 (-1.5/0.1) | ---- |
| **Basal insulin (μU.mL^-1^)** |  |  |  | |  |  |
| CTRL | 19.1±7.4 | 19.2±7.7 | ---- | | 0.01 (-0.9/1.0) | ---- |
| POL | 18.5±5.3 | 14.7±4.0 | ---- | | -0.7 (-1.6/0.1) | ---- |
| MICT | 20.1±5.8 | 18.1±7.6 | ---- | | -0.2 (-1.4/0.8) | ---- |
| HIIT | 20.5±9.2 | 19.6±8.4 | ---- | | -0.09 (-0.9/0.7) | ---- |
